# The Conservation Business

**DOI:** 10.1371/journal.pbio.0020310

**Published:** 2004-09-14

**Authors:** Henry Nicholls

## Abstract

Direct payments to local communities to conserve wildlife could prove effective but is biodiversity a commodity that can be bought and sold?

The language of conservation is changing: protecting biodiversity is no longer just about ethics and aesthetics; the latest buzzwords are commodities and consumers. Traditionally, conservation initiatives have talked up the benefits they will bring to the global community—saving species, habitats, ecosystems, and ultimately the planet. But conservation also has its costs, and these are usually borne by local people prevented from exploiting the resources around them in other ways. It is unfair to expect a localised minority to pick up costs that ultimately benefit a dispersed majority, argue conservation biologists. There has to be more money made available by concerned individuals, non-governmental organisations, national governments, and international bodies, and there need to be better ways to spend this money if conservation is to be effective, they say. Biodiversity is a commodity that can be bought and sold. We are consumers and must pay.

## Costs and Benefits

**Figure 1:**
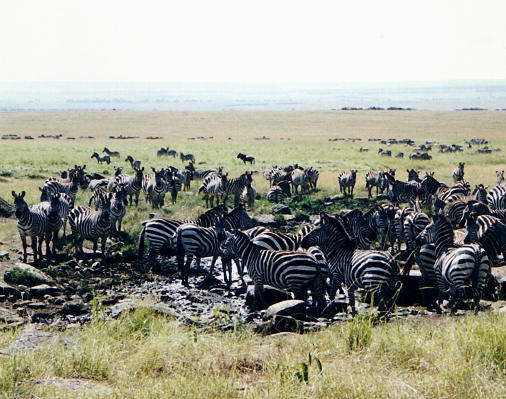
The Masai Mara National Park in Kenya Courtesy of Charlotte Stirling.

Kenya boasts one of the world's most spectacular networks of national parks and reserves covering around 60,000 km^2^ of the country (Figure 1). But devoting such a vast area to conservation has its drawbacks. It has been estimated that were this land developed it would be worth around $270 million to the Kenyan people every year. Similarly, two national parks in Madagascar are estimated to have reduced the annual income of local villagers by around 10%. Of course, protected areas do bring some benefits to neighbouring communities, most notably through tourism. But in many cases the rewards are not great, they are rarely distributed evenly among individuals, and do not necessarily outweigh the costs.

‘The costs of conservation fall disproportionately on local people, whereas the benefits are dispersed,’ says Andrew Balmford, a conservation biologist at the University of Cambridge in the United Kingdom. National and global communities stand to benefit from conservation of tropical biodiversity, but they must pay if they want to realise that benefit, he says. Conservation expenditure in the developed world is only about a third of what is needed for effective protection of 15% of the earth's terrestrial habitats, an area just large enough to preserve a representative sample of species, habitats, and ecosystems in the medium to long term (Balmford et al. 2003). The developed world must make up this funding shortfall, argues Balmford. What's more, there need to be smarter ways to spend the money that's available, he says.

## Conservation by Distraction

In recent years, many funding bodies have taken an indirect approach to conservation, investing in projects that encourage people to take up alternative practices that are compatible with conservation rather than investing in conservation itself. Perhaps the best example of this ‘conservation by distraction’ is ploughing money into community-based ecotourism projects. Such initiatives aim to bring the benefits of tourism to local people, thereby encouraging them to preserve the biodiversity they have.

It's an attractive idea. In the mid 1990s, the United States Agency for International Development was investing more than $2 billion a year in 105 conservation projects with an ecotourism component. Similarly, between 1988 and 2003, the World Bank funded 55 development projects that supported protected areas in Africa, 32 of which placed an emphasis on ecotourism.

However, an absence of quantitative data and analysis has made it hard to judge whether these projects actually achieve their dual purpose of preserving biodiversity and simultaneously reducing rural poverty. ‘Much of the information about community-based ecotourism is anecdotal and subjective,’ says Agnes Kiss of the Environment and Social Development Unit at the World Bank. The real contribution of these initiatives to biodiversity conservation is debatable, she says. ‘Many community-based ecotourism projects cited as success stories actually involve little change in existing local land- and resource-use practices, provide only modest supplement to local livelihoods, and remain dependent on external support for long periods, if not indefinitely’ (Kiss 2004).

For example, communities involved in the Infierno Community Ecotourism Project in Peru have received nearly $120,000 from their share in a tourist lodge and wages for providing services to visitors. This may have increased the income for a minority that are lodge employees, but only one family, whose adult members were all employed by the lodge, could afford to live solely on tourism. In the community as a whole, the average annual income from tourism was only $735 compared with nearly $2,000 earned elsewhere. Most of the community was still heavily dependent on other activities, and most of those activities are somewhat disruptive of conservation goals, says Kiss.

Johan du Toit of the Mammal Research Institute at the University of Pretoria in South Africa is also critical of this kind of indirect approach to conservation. At the heart of the argument for community-based ecotourism is the idea of the ‘ecologically noble savage’, he says—the notion that those living closest to nature will know what's best for it. ‘It's a wonderful idea, but it just doesn't work. Nowhere in the history of evolution has sustainability ever been naturally selected for,’ says du Toit. ‘The AK47 automatic assault rifle has replaced the bow and arrow.…Every individual in a rural community that's out hunting will shoot what he sees when he sees it, because if he doesn't somebody else will.’

**Figure 2:**
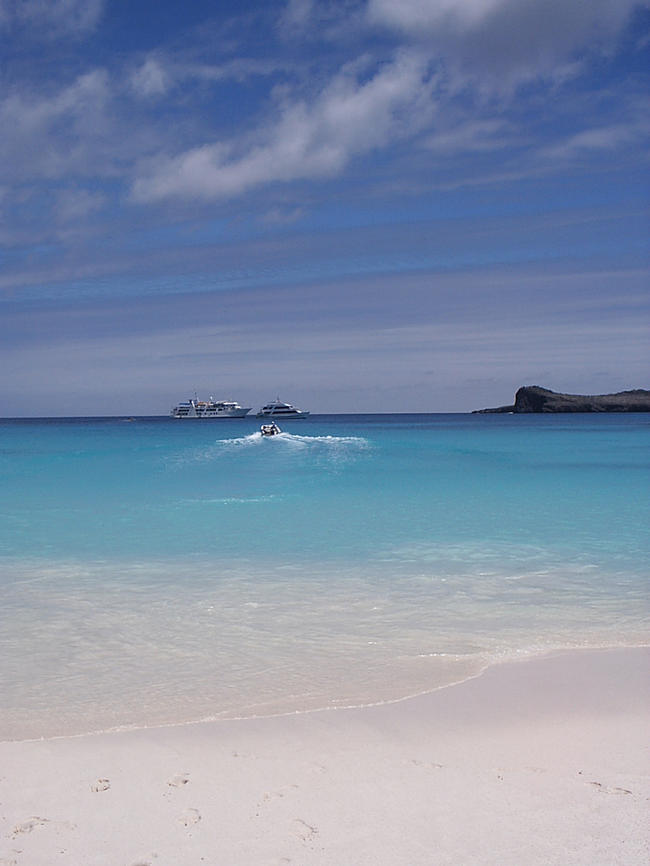
Ecotourist Paradise in the Galápagos Courtesy of Catriona MacCallum.

Nowhere is this problem more evident than in the ecotourist paradise of the Galápagos Islands (Figure 2), where a small minority of fishermen is coming into conflict with conservation aims with increasing regularity (Box 1). ‘Things are going down very quickly,’ says one Galápagos guide. ‘The iceberg is starting to tip over, and we are going to lose everything.’ If it still pays locals to exploit the environment rather than take part in one of the world's most buoyant ecotourism industries, it is clear that ecotourism alone cannot solve the world's conservation problems. Many think that ‘direct payment’ could be a useful tool. ‘Direct payment, very boldly speaking, is paying people in rural areas not to bugger up their environment,’ says du Toit. ‘It's just like if we want exclusive artworks to be looked after in the Louvre Gallery in Paris. Somebody's got to pay for it,’ he says. ‘You can't expect the Parisians who live in that arrondissement to cover the costs.’

## You Get What You Pay for—You Should Pay for What You Want to Get

For people living in developing countries, where most of the world's biodiversity exists, the short-term rewards of exploiting these natural resources are significant. Replacing indirect conservation measures, such as community-based ecotourism, with payments directly into the pockets of local people could turn out to be a much more effective way to stem this exploitation, argues Paul Ferraro, an economist at Georgia State University in Atlanta (Ferraro and Kiss 2002). It could also bring far greater development benefits than indirect financial support, he says (Box 2). An additional spin-off is that direct payments force conservation biologists to quantify and hence clarify their objectives, says John Hough, principal technical advisor on biodiversity for the United Nations Development Programme. ‘We know what we don't want,’ he says, ‘but we're not very good at saying what we do want.’

A hypothetical model simulating how Madagascar should distribute an annual conservation budget of $4 million reveals that direct payments would have protected some 80% of original forest compared with only 12% protected through a system of indirect incentives. What's more, the annual income of rural residents would have been twice that generated through indirect investment (Conrad and Ferraro 2001).

For Ferraro, the logic of direct payment is simple. He draws an analogy with a car journey from A to B. There are two routes that will bring you to B, one circuitous and the other direct. If you only have a single tank of fuel, opting for the direct route improves the likelihood you will arrive at your destination. An indirect approach to conservation is like taking the circuitous route, he says, and the chances are that you will run out of fuel. But if it's that simple, why are governments, non-governmental organisations, private bodies, and international organisations not jumping at the chance to experiment with this approach?

## Paying in Perpetuity

There are those that have reservations about direct payments. The distinction between indirect and direct interventions is artificial, says Thomas Lovejoy, president of the Heinz Center, a nonprofit institution dedicated to improving the scientific and economic foundation for environmental policy. ‘In some cases, direct payment is the only way conservation can happen,’ he says. ‘In others, the indirect is important to reinforce a situation where there already is conservation. In yet others both are needed.’

Sjaak Swart of the Section of Science and Society at Groningen University in The Netherlands argues that if conservation is to succeed, it must be rooted in the hearts and minds of those involved. Direct payments create a vision of nature dominated by calculable, monetary concerns, he says. This approach can only work in the short term, he argues, and indirect tools like debate and education are needed to involve communities in the long term. ‘You need the commitment of the local people to save the biodiversity of our world,’ he says.

Marine biologist Steve Trott agrees. He is project coordinator for the Local Ocean Trust, a charity-based conservation organisation operating in the Watamu and Malindi Marine Parks and Reserve in Kenya (http://www.watamuturtles.com), and is using direct payments to help reduce the slaughter of turtles by local fishermen. The Watamu Turtle Watch Program is currently paying fishermen just over $3 a turtle to release the animals from their nets rather than kill them. Before the scheme started in 2000, only around 50 turtles were being released from nets each year. By 2003, more than 500 a year were making it back into the sea. Elsewhere along the Kenyan coast, where fishermen do not get these payments, turtles continue to be killed, says Trott. However, the financial incentives are only part of a grander program of education and support to sensitise people to the conservation message, he says. Eventually, the plan is to stop payments altogether. ‘Payment will be reduced as education and awareness is increased to the point where it's phased out,’ he says.

Reducing or stopping the payment could work, says Ferraro, but it is more likely that the turtles will begin to suffer once more. ‘If I had to wager, I'd bet people would go back to their old patterns eventually.’ This means that direct payments require an ongoing financial commitment, and many people don't like this idea, he says.

## To the Test

The idea of direct payments needs empirical testing before it can be embraced with confidence, admits Ferraro. Funding bodies should demand experimental and control data to allow the success of an intervention to be gauged. Conservation biologists must therefore be trained in the skills needed to collect and evaluate these data. ‘Without adequate data and controls you're only going to be left with guesses and vague anecdotes about the effects of a program intervention,’ he says. Decision makers should begin to design controlled experiments from which they can make inferences about the effectiveness of these different interventions, he suggests.

There are other drawbacks of direct payments. One concern is that they might just shift the pressure from one site to another that was not previously being exploited. Furthermore, in developing countries, land tenure is often ambiguous, which can make investment an unattractive prospect for funding agencies—they want to be sure they know where their money is going. But, notes Ferraro, such objections also apply to indirect interventions. ‘I don't necessarily believe that conservation payments will be successful,’ he says. ‘It's more I believe that of all the ideas out there for protecting biodiversity, this is the least bad.’

All this talk of cost, benefit, and efficiency is creeping into conservation speak. For some, these cold and calculating terms are an odd way to describe the world's wonderfully unpredictable wildlife. But, increasingly, there are calls for conservation biology to cast aside its sentimental demons: biodiversity is a commodity that can be bought and sold; conservation is business.

**Box 1. The Cucumber Conflict**

**Figure 3:**
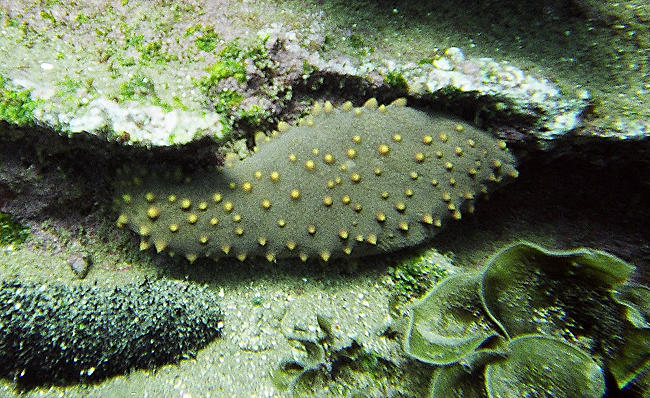
The Prized Galápagos Sea Cucumber, Stichopus fuscus Courtesy of Henry Nicholls.

At the beginning of the 1990s, fishermen in the Galápagos began collecting sea cucumbers from the waters around their islands to meet ongoing demand for these aphrodisiac ‘earthworms of the sea’ in Southeast Asia (Figure 3). Others intent on taking advantage of this commercial opportunity began to arrive from the Ecuadorian mainland in their hundreds. In 1998, Ecuador's president signed the Special Law of the Galápagos, which created the Galápagos Marine Reserve, protecting its waters from commercial fishing and imposed restrictions on domestic immigration. But by then, too many were already intent on reaping the financial rewards the sea cucumber promised them—by the end of the decade, a single sea cucumber could fetch nearly $2. Conservation biologists at the Charles Darwin Research Station on the central island of Santa Cruz worked out levels of fishing that might be sustainable. In 1999—the first season in which sea cucumber fisheries were monitored and regulated—nearly 800 fishermen collected more than 4 million animals worth more than $3.4 million in a short two-month window. In January 2000, fishermen protesting the closure of the fishery took over offices of the Galápagos National Park Service and Charles Darwin Research Station, holding humans and animals hostage.

**Box 2. Paying for Forests**

**Figure 4:**
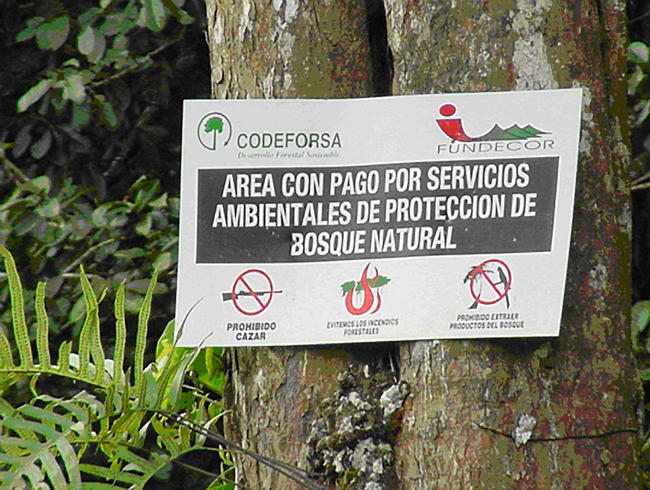
Forest Protected by Costa Rica's PSA Courtesy of Subhrendu Pattanayak.

The longest-running and best-known example of a direct-payment initiative is the Programa de Pago de Servicios Ambientales (PSA) in Costa Rica (Figure 4). In the second half of the 20th century, forest cover in Costa Rica fell from around 50% to 25%, and more than half of what remained was on unprotected, privately owned land. In 1996, the PSA was set up to make direct payments to individual landowners, associations of landowners, or indigenous reserves in exchange for ‘environmental services’—anything from forest conservation to providing a supply of water. Some 85% of contracts have been for forest conservation. By 2001, more than 2,800 km^2^ were protected by payments of $4,000 per km^2^ every year, and contracts covering a further 8,000 km^2^ were being processed. Most of the money for these payments comes from a petrol tax on Costa Rican citizens, although the Global Environmental Facility has put up money for biodiversity conservation, Costa Rica's Office of Joint Implementation has paid for carbon sequestration, and domestic hydroelectricity and municipal water providers pay for water services.

## Abbreviation

PSA: Programa de Pago de Servicios Ambientales

## References

[pbio-0020310-Balmford1] Balmford A, Whitten T (2004). Who should pay for tropical conservation and how the costs should be met. Oryx.

[pbio-0020310-Balmford2] Balmford A, Gaston KJ, Blyth S, James A, Kapos V (2003). Global variation in terrestrial conservation costs, conservation benefits, and unmet conservation needs. Proc Natl Acad Sci U S A.

[pbio-0020310-Conrad1] Conrad JC, Ferraro PJ (2001). Habitat conservation: The dynamics of direct and indirect payments. Environmental Policy Working Paper Ser.

[pbio-0020310-duToit1] du Toit JT, Walker BH, Campbell BM (2004). Conserving tropical nature: Current challenges for ecologists. Trends Ecol Evol.

[pbio-0020310-Ferraro1] Ferraro PJ, Kiss A (2002). Direct payments to conserve biodiversity. Science.

[pbio-0020310-Ferraro2] Ferraro PJ, Simpson RD (2004). Protecting forests and biodiversity: Are investments in eco-friendly production activities the best way to protect endangered species and enhance rural livelihoods?. Paper presented at the International Conference on Rural Livelihoods, Forests and Biodiversity.

[pbio-0020310-Kiss1] Kiss A (2004). Is community-based ecotourism a good use of biodiversity conservation funds?. Trends Ecol Evol.

[pbio-0020310-Swart1] Swart JAA (2003). Will direct payments help biodiversity?. Science.

